# Health-system drivers influencing the continuum of care linkages for low-birth-weight infants at the different care levels in Ghana

**DOI:** 10.1186/s12887-023-04330-5

**Published:** 2023-10-05

**Authors:** Christina Schuler, Faith Agbozo, George Edward Ntow, Veronika Waldboth

**Affiliations:** 1 School of Health Sciences, Institute of Nursing, Zurich University of Applied Sciences (ZHAW), Winterthur, Switzerland; 2 FN Binka School of Public Health, Department of Family and Community Health, University of Health and Allied Sciences, Ho, Ghana; 3 Dodowa Health Research Centre, Dodowa, Ghana

**Keywords:** Continuum of care, Coordination of care, Delivery of health care, Ghana, Constructivist grounded theory, Low birth weight, Newborn care, Neonatal nursing

## Abstract

**Background:**

Low birth weight (LBW) is associated with short and long-term consequences including neonatal mortality and disability. Effective linkages in the continuum of care (CoC) for newborns at the health facility, community (primary care) and home care levels have a high tendency of minimizing adverse events associated with LBW. But it is unclear how these linkages work and what factors influence the CoC process in Ghana as literature is scarce on the views of health professionals and families of LBW infants regarding the CoC. Therefore, this study elicited the drivers influencing the CoC for LBW infants in Ghana and how linkages in the CoC could be strengthened to optimize quality of care.

**Methods:**

A constructivist grounded theory study design was used. Data was collected between September 2020 to February 2021. A total of 25 interviews were conducted with 11 family members of LBW infants born in a secondary referral hospital in Ghana, 9 healthcare professionals and 7 healthcare managers. Audio recordings were transcribed verbatim, analyzed using initial and focused coding. Constant comparative techniques, theoretical memos, and diagramming were employed until theoretical saturation was determined.

**Results:**

Emerging from the analysis was a theoretical model describing ten major themes along the care continuum for LBW infants, broadly categorized into health systems and family-systems drivers. In this paper, we focused on the former. Discharge, review, and referral systems were neither well-structured nor properly coordinated. Efficient dissemination and implementation of guidelines and supportive supervision contributed to higher staff motivation while insufficient investments and coordination of care activities limited training opportunities and human resource. A smooth transition between care levels is hampered by procedural, administrative, logistics, infrastructural and socio-economic barriers.

**Conclusion:**

A coordinated care process established on effective communication across different care levels, referral planning, staff supervision, decreased staff shuffling, routine in-service training, staff motivation and institutional commitment are necessary to achieve an effective care continuum for LBW infants and their families.

**Supplementary Information:**

The online version contains supplementary material available at 10.1186/s12887-023-04330-5.

## Background

Globally, more than 20 million babies are born low birth weight (LBW) (< 2500g) each year [86]. LBW prevalence in Africa is high (13.7%) with majority of LBW infants born in Eastern and Western Africa [86]. Ghana is no exception with a LBW rate of 14.2% [80].

LBW results from premature birth, small-for-gestational age or both [39] with short- and long-term health consequences [42]. Short term complications include neonatal and post-neonatal deaths arising from respiratory problems, infections [58], growth failure and developmental delay [48, 63]. Later in life, LBW is likely to lead to low intelligence quotient and adult-onset of non-communicable diseases such as diabetes and coronary heart disease [23, 38, 41]. LBW coupled with poor neonatal services have a high impact on socio-economic burden for families, health systems and national budgets [13, 35, 58].

In order to improve newborn health outcomes and reduce economic burden, high quality and standardized care rendered through a structured and well-coordinated Continuum of Care (CoC) is vital [11, 87]. In low-resource settings, health services are often fragmented with no specific post-discharge referral system for LBW infants who require additional post-hospitalization care [27, 77]. Addressing this fragmentation requires well-coordinated CoC that links hospital care to both community (primary) and home care. This will reduce patients’ tendency of receiving suboptimum care that results from poor connection within the CoC [44].

The concept of CoC involving integrated services and the appropriate personnel to deliver services was first described by Liebowitz and Brody [57]. Kerber et al. [44] adapted the CoC for Maternal, Newborn and Child Health (MNCH) services in the context of resource constraint countries by linking pregnancy, childbirth, and postpartum support at different care levels encompassing facility, community and home care [44]. Different MNCH services are often not effectively linked to each other within the health system [44–46]. The fragmentation is most prominent in the postnatal care with the biggest negative impact on the most vulnerable such as LBW infants [85]. The MNCH care continuum is affected by economic, cultural and social factors making the provision of an effective CoC challenging for many countries, including Ghana [45, 46, 66].

To achieve a quality CoC that contributes to reducing neonatal mortality and disability, the Ghana Ministry of Health has put in several measures to strengthen its MNCH healthcare system. In 1998, the Integrated Management of Childhood Illnesses (IMCI) approach was introduced in Ghana [16] with the aim to reduce morbidity and mortality among children under the age of five [89]. Two years later, the Community-based Health Planning and Services (CHPS) was established to improve access to primary healthcare and empower underserved communities in health governance [20, 50]. In 2005, the National Health Insurance Scheme (NHIS) complemented the cascade of services. Since 2008, the NHIS has offered mothers access to free health care service in the antenatal, intrapartum and postnatal period [9, 64, 65]. Neonates are covered for free under the mother’s health insurance in the first three months of life [65]. After the first three months of life, children are eligible for free essential healthcare services until they reach 18 years of age. They can register for this coverage independently, and are only required to pay a nominal processing fee [52, 65]. Introduction of the NHIS and the free basic health care for infants has significantly contributed towards reducing neonatal mortality [52]. Another initiative to improve the health outcomes of small and sick newborns in Ghana was the implementation of Every Newborn Action Plan (ENAP) in 2014. Despite these investments, Ghana still faces many challenges in achieving continuity and quality in healthcare [66]. Single components of MNCH services have been studied but the care for LBW infants as a continuum in Ghana has not gotten much attention [43, 92] as data is dearth on the CoC and the critical transition of LBW infants within the different levels of care [2, 34, 66]. Assessing the current postnatal care continuum can identify gaps and enabling factors that influence the linkages and transition between the different care services in Ghana’s health system.

Thus, the objective of this study was to gain in-depth insight into the drivers within the health care system in Ghana which influences the continuum of care linkages for low birth weight (LBW) infants at the different levels of care. In achieving this objective, particular attention is paid to the organizational aspects, processes, functions, as well as the experiences and needs of individuals involved in the care process for LBW infants.

The following research questions guided the study: What drivers influence the continuum of care linkages for LBW infants in Ghana? How is the organization of the care transition for LBW infants managed from regional hospitals (secondary level of care) to communities and home care (primary level of care) settings and which health professionals and stakeholders participate in the care transition process in Ghana?

## Method

### Design

The focus of this study was to move beyond identifying and describing the CoC for LBW infants in Ghana by generating a theoretical model that deepens the understanding of “what and why it is going on” [30]. Charmaz’s [25] qualitative constructivist grounded theory approach was considered an ideal methodology to identify key processes in the CoC including experiences and needs of both health providers and families of LBW infants. This design recognizes the way in which the interaction between the researcher and participant can influence data construction [25] and investigates complex phenomenon such as demand and provider-side perspectives in health service delivery [29].

### Setting

The study took place in a municipality in Ghana having a regional hospital, several primary-level health centers and CHPS zones serving over 200.000 inhabitants. The hospital provides emergency obstetric and essential newborn care as well as caring for sick and small newborns at the Neonatal Intensive Care Unit (NICU). Key services offered at the NICU include neonatal resuscitation, oxygen therapy, phototherapy, nasogastric tube feeding, treatment of neonatal infections and kangaroo mother care. The average number of small and sick newborns cared for in the NICU per month is 39. At the time of data collection, the NICU was staffed with eight general nurses, one pediatric specialist nurse, one pediatric physician and one general medical officer. Within the community, the CHPS provide basic MNCH services and adult health services. Neonatal care includes support for breastfeeding, growth monitoring, vaccination, management of minor ailments and referrals to higher-level facilities [61]. The CHPS zones are run by community health nurses sometimes complemented by midwives and physician assistants. Majority of the population in the study setting are into petty trading, crop farming and livestock keeping.

### Participant selection

The initial sampling method entailed defining sampling criteria for potential participants. Criterion sampling was used purposefully to select participants who met the prespecified eligibility criteria [29, 71]. There were two groups of participants with the first being healthcare professionals (HCPs) who provided direct care either at the hospital and community level and healthcare managers (HCMs) in managerial and administrative positions at the hospital, district, and regional levels. HCPs who were still undergoing basic training or had been in their current position for less than one month were not considered eligible. The second group included mothers and relatives whose LBW infants were admitted for at least 24 h to the NICU and later discharged home not longer than eight weeks prior to the interview. The time frame of 24 h was chosen because in LMICs, healthy newborns, including LBW infants are often discharged early due to overcrowded neonatal units, mothers’ having older children at home to care for, lack of support and financial constraints faced by parents [4, 24, 35]. The mothers and relatives of LBW infants who had been transferred to a higher-level care facility after being admitted at the NICU and had not returned to the NICU were not recruited.

Relatives other than mothers and fathers are often substantially involved in the care of LBW infants in Ghana [3, 77]. Therefore, other caregivers pointed out by the mothers to be significantly involved in caregiving for their LBW infants were also recruited.

The caregivers in this study were purposively sampled without regard to their educational status. All participants were required to speak the local language (Ewe) or English.

After the initial contact was established through a gatekeeper, interviews were first conducted with the HCPs at the NICU. Further interviews were held with facility-based HCPs directly involved in clinical care and HCMs responsible for managerial tasks concerning LBW infants. Later, HCPs from four CHPS zones at the primary care level and HCMs from the district and regional levels were interviewed. All interviews with HCPs and HCMs were conducted in English. Families of LBW infants were called and informed about the study. After agreeing to participate, those unable to speak English were interviewed in the local language. All prospective participants who were contacted consented with none unwilling to participate nor withdrawing the consent along the line. After initial sampling theoretical sampling followed. Theoretical sampling is an open selection of data that helps to decide what subsequent data to collect to develop a theory [28, 37]. Subsequently, additional HCPs and HMCs at different levels of care were recruited after earlier interviews revealed that they played a major role in the care continuum. Sampling continued as theoretical categories were developing. This involved selecting new participants or re-interviewing earlier participants. For example, to expand the category concerning the discharge process, two additional health professionals at the hospital and another two at community level were interviewed. The follow up interviews helped to specify the properties of selected categories and to get more in-depth information where details were still lacking. Theoretical saturation was reached, when new data collected did not reveal any new properties to the theoretical categories nor provided any further insights about the emerging theory [25].

### Data collection and analysis

Data collection and analysis started simultaneously in September 2020 and was concluded in April 2021. Participants perspectives on their personal experiences with the study topic were explored through intensive interviewing. Intensive interviewing is a gently guided open-ended, one-sided conversation style, which is used in constructivist grounded theory [25]. For each participant group an interview guide comprising themes, a few open-ended and probing questions were developed. Two of the four interview guides (Supplementary data 1, 2, 3 and 4) were pretested with a neonatal nurse and with both a mother and a father, who were interviewed separately in a neighboring municipality. The interview guides for HPCs at primary care level and for health care managers were similar. They did not need pretesting as they had been adapted based on the experience gathered from the pretesting of the other interview guides. Apart from four interviews with the healthcare managers where only the first author was present, all interviews were conducted by the first and third authors. The interviews started with broad open-ended questions inviting interviewees to narrate their experiences and views concerning the care of LBW infants. Probing questions provided in-depth information as well as uncovered hidden actions and practices and their implications on the care continuum. An example of a question for health professionals was: “can you tell me how it is for you to provide care for LBW infants here in NICU/community”. A question for healthcare managers: “can you tell me ways in which you are engaged in the planning and implementation of care for LBW infants at your institution and/or district/regional level?” A typical question for caregivers was: “can you tell me how you experienced your time in the NICU?”.

In the later stage, focused questions generated a more detailed discussion about the topic [25]. Interviews were conducted in the participant’s place of choice ranging from the NICU, the HCMs offices, CHPS compounds to the family’s home. The audio-recorded interviews lasted between 30 to 111 min with an average of 56 min. Each interview, including those conducted in the local language, was directly transcribed verbatim in English using the f4transcription program [32]. An independent person fluent in both languages made spot checks on the transcripts to ensure quality of the language translation. Transcripts of all English interviews were counterchecked using dual control principle to ensure quality and rule out mistakes [33] before storing on a secured platform. Sociodemographic information was gathered to provide an overview of the participants. Descriptive analysis of the sociodemographic information was conducted to describe the study population. Frequencies were computed using Microsoft Excel. Immediately after each interview, observations and experiences of the interviewee were documented in field notes which were later used in memo writing. Data analysis entailed an iterative process that generated a theoretical explanation of the CoC built on the views and experiences from both the demand and supply side. Throughout the data analysis, the research team held regular meetings to discuss the analysis process. Data was analyzed inductively using Atlas.ti version 9.2. Initially, line by line coding and constant comparison techniques were applied [36]. Although the coding process was guided by the research questions, the authors remained open to explore alternative analytical options. The codes that assisted in addressing the research questions and enriching the comprehension were progressively pursued. Focused coding followed to verify the adequacy and conceptual strength of the initial codes. The selected focused codes were used to develop categories thereby advancing from a pure descriptive level of analysis to a more abstract, theoretical level [25]. To verify and saturate the codes and emerging categories, the technique of sorting and diagramming was used in addition to theoretical coding. Field notes and memo writing assisted further to describe the results and develop the theoretical model. Relationships between the categories were delineated using ATLAS.ti’s network function which helped to examine relations between the different actors and processes [60]. Data collection ceased when the categories were insightful, demonstrated analytical precision and established relationships between the categories.

### Rigor

Charmaz’s [25, 26] quality criteria for grounded theory studies were applied to ensure rigor during the design, data collection, analysis and assessment of strengths and limitations of the study. The credibility criterion requires sufficient familiarity with the study setting, use of relevant data collection tools and the researchers’ continuous reflexivity. An established collaboration and a profound understanding of the study settings by the local partners and years of work experience by the first author facilitated the achievement of credibility. Sufficient relevant data and making systematic comparisons throughout the research process contributed to meeting this criterion. Through a continuous reflective practice with the research team and transparent, detailed report on the methodological procedures and study findings, contextual sensitivity was achieved. The use of field notes added to the thick description of the research context [72]. Originality involves the portraits of new insights. Original insight into individual understanding were sought from both the health professionals and families about the CoC of LBW infants to enhance knowledge in the field of neonatal care in Ghana and in similar contexts. Resonance asks for the fullness of studied experiences. To ensure resonance, data analysis and findings relied on member checking. Preliminary results and the theoretical model were checked with one participant each from the hospital and the community for reflection and accreditation. Because personal experiences might not be recognized in synthesized findings and some memories can cause distress among participants [21], preliminary findings were not shared with family member. Usefulness implies applicability of findings for practice. Usefulness of the study towards advancing knowledge and optimizing care for families with LBW infants was assessed by presenting the results to the health professional followed by a discussion and feedback on how to improve patient care before study completion.

### Ethical consideration

Ethical clearance was obtained from the Ghana Health Service Ethical Review Committee (GHS-ERC 029/07/20). The regional and district health directorates as well as the hospital management granted permission to conduct the study. At the first contact, participants received brief information on the study either through face-to-face or via telephone conversations and their verbal affirmation served as evidence of willingness to participate. Before the interviews, the information sheet was read aloud to participants who could not read English in the presence of a witness, after which the participants thumb-printed or signed the consent sheet. After each interview, audio files were encrypted and stored safely together with field notes to safeguard confidentiality of the participants.

## Results

### Socio-demographic characteristics of participants

The 27 participants interviewed in 25 interviews comprised 16 (59.3%) health professionals and the remaining 11 (40.7%) were family members who cared for LBW infants. The health professionals consisted of HCPs who provide direct care either at the hospital (*n* = 5) or community level (*n* = 4) and healthcare managers (HCMs) in administrative positions at the hospital (*n* = 3), district (*n* = 2), and regional levels (*n* = 2). Among the health professionals, there was one pediatrician and one pediatric nurse. Majority of the health professionals (43.8%) were within the age group of 51- 60 years with an overall mean age of 43.1 years (± 11.0 years) and more than half (56.2%) of the health professionals were females. The second group included mothers (*n* = 7) and relatives (*n* = 4) comprising a father, an uncle, an aunt, and a grandmother. The predominant age group for the mothers was the 20–25 years age group (42.9%) with none above the age of 40. Family members’ age ranged from 32 to 46 years with mean age of 37 ± 7.0 years. The mean age of the LBW infants at the interview was 40.6 ± 9.1 days. Coincidentally, all the LBW infants whose guardians were included in this study were female babies. Tables 1, 2 and 3 show the socio-demographic characteristics of participants.

**Table 1:** Sociodemographic characteristics of healthcare professionals

**Health professionals *n* = 16**		
**Variable**	**Groups**	**Number (%)**
Sex	Male	7(43.8)
	Female	9(56.2)
Age (years)	< 30	1(6.2)
	30—40	6(37.5)
	41—50	2(12.5)
	51—60	7(43.8)
Direct Care	NICU	3(18.8)
	PHNU	2(12.5)
	CHPS	4(24.9)
Indirect Care/Management	Hospital level	3(18.8)
	District level	2(12.5)
	Regional level	2(12.5)
Highest Educational Level	Certificate	2(12.5)
	Diploma	7(43.8)
	Bachelor	2(12.5)
	Masters	5(31.2)
Years of experience	0—5	1(6.2)
	6—10	6(37.5)
	11—15	2(12.5)
	20—30	5(31.3)
	31—40	2(12.5)
Years in the current position	< 1	3(18.8)
	1—5	10(62.5)
	6—10	2(12.5)
	11—15	1(6.2)

*Abbreviations:* *n* Number, *NICU* Neonatal Intensive Care Unit, *PHNU* Public Health and Nutrition Unit, *CHPS* Community-based Health Planning and Services

**Table 2:** Socio-demographic characteristics of the mothers and their family relations

**Mothers *n* = 7**			**Relatives *n* = 4**
**Variable**	**Groups**	**Number (%)**	**Number (%)**
Sex	Female	7(100)	2(50)
	Male	0(0)	2(50)
Age	20—25	3(42.9)	0(0.0)
	26—30	2(28.5)	0(0.0)
	31—35	1(14.3)	2(50.0)
	36—40	1(14.3)	1(25.0)
	41—45	0(0.0)	0(0.0)
	46—50	0(0.0))	1(25.0)
Education	Primary	1(14.3)	0(0.0)
	Junior High School	3(42.9)	2(50.0)
	Senior High School	2(28.5)	0(0.0)
	Tertiary	1(14.3)	2(50.0)
Employment	Informal	3(42.9)	2(50.0)
	Formal	1(14.2)	1(25.0)
	Unemployed	3(42.9)	1(25.0)
Marital status	Single	2(31.6)	0(0.0)
	Married	5(68.4)	3(75.0)
	Widowed	0(0.0)	1(25.0)
Place of residence	Semi-urban	5(47.4)	3(75.0)
	Rural	2(52.6)	1(25.0)
Religion	Muslim	1(14.3)	1(25.0)
	Christian	6(85.7)	3(75.0)

**Table 3:** Sociodemographic of the low-birth weight (LBW) infants

**Low birth weight infants *n* = 7**		
**Variable**	**Groups**	**Number (%)**
Sex	Male	0(0.0)
	Female	7(100.0)
Age of child at interview (days)	26—30	2(28.6)
	31—35	0(0.0)
	36—40	2(28.6)
	41—45	1(14.3)
	46—50	1(14.3)
	51—60	1(14.3)
Birth order	1^st^ child	2(28.6)
	2^nd^ child	1(14.3)
	3^rd^ child	4(57.1)
Gestational age at birth (weeks)	32—33	2(28.6)
	34—35	2(28.6)
	36—38	3(42.9)
Birth weight (kg)	1.0—1.4	2(28.6)
	1.5—2.0	4(57.1)
	2.1—2.5	1(14.3)
Time spent at NICU (days)	0—7	3(42.9)
	16—20	3(42.9)
	21—30	1(14.2)
Time spent at home after discharge (days)	11—20	2(28.5)
	21—30	1(14.3)
	31—40	3(42.9)
	41—50	1(14.3)

*Abbreviation:* *NICU* Neonatal Intensive Care Unit

### Factors influencing the continuum of care for LBW infants

**Fig. 1:**
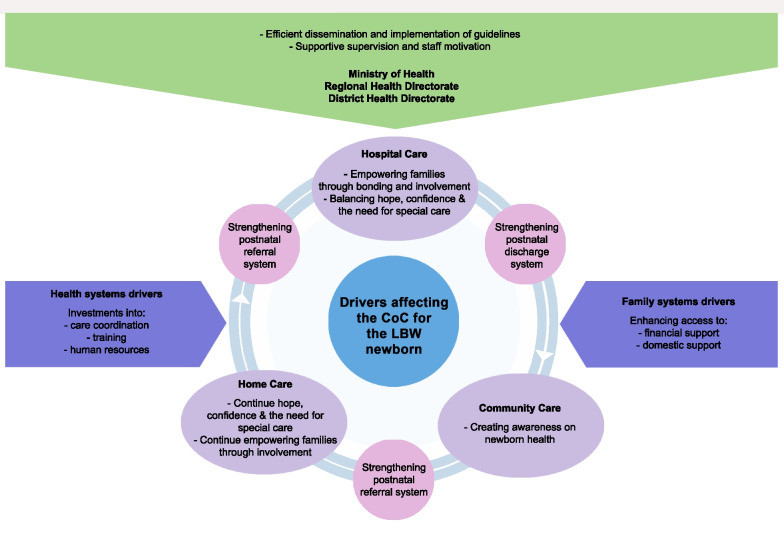
Care continuum for LBW infants in Ghana detailing inputs at the different care levels [78]

After analyzing the interviews, we developed a theoretical model (Fig. 1) that explains the structures and processes underpinning the care continuum for LBW newborns in Ghana. The theoretical model, described from ten major themes, also highlights the experiences and needs of families and health professionals regarding the CoC. The themes were broadly categorized into family-system and health system drivers. The four themes related to family-system drivers are described elsewhere [78]. In this paper, we focus on the six health systems drivers: strengthening 1) postnatal discharge; 2) review; 3) referral system; 4) efficient dissemination and implementation of guidelines; 5) supportive supervision and staff motivation and lastly; 6) investments into care coordination, training and human resource. The CoC is complemented by including the outer context which influences decisions and actions on CoC, that is, the District and Regional Health Directorates, and the Ministry of Health.

Ideally, care for LBW infants should begin in the hospital and, where LBW is detected in utero, care should be initiated during the antenatal period. After delivery, this care transitions to the community (primary) level and continues at home. Families return to the primary or secondary facility for review check-ups but in case of deteriorating health, LBW infants are referred to the hospital care making it a continuous circuit of care. In the ensuing sections, we discuss the different drivers which positively or negatively influence the transition within the different care levels.

### Strengthening postnatal discharge system

The health professionals identified fragmentation in the discharge pathway between the different levels of care, which posed a risk for LBW infants in the critical postnatal phase:

“One of the challenges now that we are trying to work on is the apparent division. (..) The two [Hospital and community care] should overlap because health care delivery does not end with hospital discharge. So one challenge is making sure that this transition is smoothly done between hospital and community” [HCM hospital]

The HCM noted the importance of increasing awareness that both clinical and community care are needed to achieve comprehensive care along the patient pathway thus fostered collaborations between the different levels of care*.* However, continuity of care from the hospital to primary care to the home was hampered by an unstructured discharge pathway. Sometimes, the public health and nutrition unit (PHNU) located in the hospital, linked families of LBW infants with the CHPS zone before discharged home from the NICU. Instead of referring LBW infants to the CHPS zones after discharge, often, the PHNU maintained contact with the families via phone calls without referring. Occasionally, families by themselves reported to the CHPS zone after discharge. Meanwhile, not all families were informed concerning the nearest CHPS zone or when to begin child welfare sessions:

“As for the baby at this age, I don’t know. (…) Maybe within this month or next month actually before they will direct her (the mother) to any place to go for the weighing.” [Family member, birth weight (BW) 1.35 kg, gestational age (GA) 35 weeks]

Sometimes the NICU liaised directly with the CHPS via referral letters or calls. But community HCPs were not always informed about the discharge. To work efficiently, whenever a LBW infant is referred, community HCPs expressed desire to receive explicit care instructions together with a medical history. A HCM explained why clear care instructions are necessary to guide community HCPs who lacked specialized knowledge:

“They [CHPS staff] don’t have the specialized training (..), any time you want them to do something specific, you have to give them specific instructions on what you expect them to do.” [HCM hospital]

Community HCPs mostly found out about LBW cases at child welfare clinic sessions when mothers brought their babies for their first vaccinations or when community volunteers identified the LBW infants in the community notified the HCPs. One HCP explained how they supported volunteers to identify LBW infants:

“What we have been doing is that we give them [community volunteer] information that anytime we have babies of that, they should let us know. (….) So when when they are moving around, they hear that somebody has delivered. (…..) We even show him where to check to see whether this baby is below 2.5 [kg], and [they] let us know.” [HCP CHPS]

Although the community volunteers’ work was voluntary, their community is supposed to give them support for their activities as motivation so they can have adequate time to carry out the voluntary work. But according to a district HCM, community support has reduced, thus community volunteerism has become less active. In few instances, traditional birth attendants closely collaborated with the CHPS facility and worked concertedly during the intrapartum period. But generally, HCPs did not collaborate with or promote the involvement of traditional birth attendants as the latter are seen to lack adequate knowledge about LBW infants.

Home visits were perceived as important by all cadres of health professionals to reinforce educational messages, involve families, assess the client’s general health condition and where necessary, initiate hospital referral. Thus, home visits were often conducted when community HCPs were aware of the presence of LBW infants. However, due to understaffing and a lack of funds for transportation, frequent home visits were limited to communities closer in proximity rather than extending to remote villages. Except for one mother who received antenatal care at the CHPS compound and was contacted by a community-based HCP during the postnatal period, families that participated in this study did not receive any home visits.

### Strengthening postnatal review system

Health professionals held different views about the review procedure after birth where LBW infants are supposed to come back to the hospital for developmental and health check-ups. Generally, families returned to the hospital on scheduled dates for review and the baby’s development, overall wellbeing, weight, breastfeeding, temperature, and occasionally, oxygen levels were assessed. Meanwhile, some mothers did not perceive these reviews as very informative, as they did not understand what was done during the review visits nor could they ask questions:

“I just heard the one who weighed the baby was telling the one who was recording that it was 1.9 [kg]. But for counselling or education, they didn’t tell me.” [Mother, BW 1.7 kg, GA 36]

Some health workers thought the review system was not yet well established. Financial problems and long travels were major barriers for some families to attend scheduled review visits forcing some to abandon their appointment or opt to attend the CHPS compound instead. According to the hospital HCPs, assessing developmental milestones should continue for at least one year. But the visits were often discontinued earlier due to insufficient human capacity and space to carry out the check-ups.

To strengthen the review system, families had to leave their phone numbers, so they could be contacted in case they missed the scheduled review visit. Some HCPs at the NICU and PHNU sometimes attempted calling families to enquire about their wellbeing, but provision of non-functional phone numbers and network instability made it difficult reaching families. HCPs at the NICU had to use their personal money for the calls because no institutional provisions were made.

“That one [review visits], let me say for our side here, we are very poor in that. (…) Because when you discharge a client and some of them refuse to come [back], we don’t do follow up to find out the reason why the person is not coming.” [HCP NICU]

### Strengthening postnatal referral system

Staff at the CHPS compound often lacked expert knowledge concerning the care of LBW infants and CHPS facilities were not equipped to treat LBW infants. Thus, when LBW infants fell sick, they had to be referred to a higher-level hospital. Financial issues and fear of visiting the hospital due to negative experiences sometimes led families to avoid hospital referrals. As this HCP explained, families often did not understand why their LBW infant cannot be treated at the CHPS compound like other babies:

“They just do not understand it, ahaa. Why do you attend to other babies but my own, because of low birth weight, you said we should go to the hospital. When you mention hospital, then wahaala [A slang word meaning trouble]. Is it hell for them. (…) So if you don’t ensure that they pick the car in your presence, forget it, they won’t go.” [HCP CHPS]

To ensure families comply with the referral, staff at the primary level occasionally accompanied families to the hospital with the health staff occasionally bearing the cost. Barriers complicating prompt referral process include unstable phone networks, difficulty arranging transportation especially at night, and uncoordinated referral communication whereby the NICU is uninformed in advance about the referral. High transportation costs and lack of pediatric ambulance were additional barriers for referral back to the hospital or to higher level facilities.

Families did not have phone numbers of the hospital where they could call for advice when they experienced deteriorating health or had question concerning the care of their LBW infants. Although families were taught common neonatal danger signs, when something was unusual, they either waited for it to be resolved or bought over-the-counter medicines instead of seeking health care from the hospital:

“One day we saw that the side of the vagina was hard, so they [drugstore] showed us some medicine and we use it on it then the the thing went off.” [Mother, BW 2.4 kg, GA 38]

### Efficient dissemination and implementation of guidelines

Clinical care of LBW infants and managerial activities such as supervision, evaluation and resource allocation were guided by the ENAP framework but frontline workers at the primary level were unaware of the action plan:

“The unfortunate aspect is the dissemination of that [ENAP] involves only senior managers from either the regional level and the national level and then the district level. But those who are at the lower level, who are actually doing the work, they may not have so much access and knowledge about”. [HCM region]

HCMs emphasized that all health workers irrespective of their level of care should be informed about the ENAP as enhanced dissemination creates awareness and commitment for newborns at all levels of care. HCMs suggested that supervisory visits and trainings could be a means of linking care activities with the ENAP to maintain the same standard of care throughout the CoC. Staff at the NICU had access to guidelines concerning treatment of conditions such as prematurity, birth asphyxia and neonatal infection which generated a sense of security. Staff who had a specialized training in pediatrics were more familiar with the guidelines and protocols and thus had more in-depth knowledge about nurturing neonatal care. However, health professionals at the CHPS and PHNU had limited access to such guidelines which could better regulate their care provision and improve quality of care:

“Low birth weight babies. (…) Are there some [guidelines]? Is there a written document on their management? (…) Maybe you are doing something which does not conform to that guideline, then you take caution. [HCP PHNU]

### Supportive supervision and staff motivation

Staff supervision was done at every level of care, but opinions differed on its effectiveness. Supervision was seen as a tool to receive feedback, motivate and appraise staff performance, and make corrections through on-the-job training:

“Sometimes they cannot get everything right so when we go, we support them, check on them, guide them or mentor them on how to do things right so they can also replicate this.” [HCM district]

Staff understood the role of supervision in improving the work situation at the lower level. Some hospital and community-based staff desired that HCMs visited their units more often to ascertain their work-related challenges:

“All boils down to the supervision. They are not coming down to see the realities. (..) That is the problem. (…) If the person really comes to the ground to see. (…) They would understand that ohh you need this, you need this.” [HCP NICU]

Staff felt unheard when their challenges (particularly lack of equipment and inadequate infrastructure) were not addressed. A HCP complained about not having seen any changes of the work condition in her years of service:

“A lot of things need to be changed. But you appeal, nobody is willing. (…) They keep promising, authorities keep promising but, for my six and half years that I have been here, nothing has been done.” [HCP CHPS]

Management was unable to meet their needs at all or on time due to laxity of the health insurance system in reimbursing their claims on time.

Staff motivation was an enabling factor in achieving good quality care, the most significant contributors being healthy clients and satisfied families. HCPs in the hospital and CHPS improvised to provide best possible care:

“The motivation actually is your client. (…) So irrespective of the non-availability of those things [equipement] we are motivated when you try your best, using some improvised method and the babies are fine.” [HCP NICU]

The positive health outcomes for LBW infants were a source of happiness and satisfaction for the staff. But they were often understaffed which hindered their ability to fulfill basic duties such as making follow-up calls and conducting home visits. This together with the constant lack of equipment resulted in frustration and loss of motivation to work. Despite these challenges, some HCPs showed high work moral by personally purchasing workplace equipment occasionally and supporting mothers when they had little to feed. Financial incentives from HCMs and the passion to care for vulnerable babies was a driving force.

### Investments into care coordination, training and human resource

Opinions of HCMs varied regarding knowledge on neonatal care. While some were satisfied, others felt knowledge on LBW was lacking or outdated. HCPs, especially those working at the primary level were not satisfied with their knowledge and competencies and wished for more training opportunities. The annual reshuffling of staff from one ward to another affected knowledge retention and quality of care. To avoid the knowledge loss, one HCP suggested:

“So, you can enhance it by not moving them [nurses] round like a chess game. (..) Every year then they reshuffle them. Then at one station they learn a little bit then they move. But for the neonatology, we need more on the job training and longer stay before they can do the job.” [HCP NICU]

HCMs explained that the challenge has shifted from neonatal survival to appropriate neonatal care for the survivors, but the investments were not well-structured hampering care planning and provision:

“The care when the baby survives is the challenge now. The human resource challenge that we have now, because the investment has not been well-structured and organized. (…) When the knowledge is well assimilated and put into practice, then we could see some improvement. (….) That’s why I am saying that we have not done enough investment.” [HCM region]

Restructuring newborn care emphasizing LBW and preterm care was suggested by HCMs at both district and regional levels. Regarding facilitating factors, strong leadership commitment and stakeholder involvement at all levels of care emerged. Willingness to strengthen newborn care at the political level and successfully implement policies and guidelines such as the ENAP at lower levels of healthcare were further factors which were mentioned by the HCM to achieve quality care for LBW infants.

## Discussion

This constructivist grounded theory study provides in-depth insights into the experiences and needs of both healthcare professionals and families concerning the structure, processes and persons involved in the CoC for LBW newborns in the neonatal period in Ghana as well as the challenges and enabling factors. Receiving views from both the demand and supply side as part of our research approach has contributed important new insights to the scarce literature concerning the CoC for LBW infants and their families. These findings can inform future improvements in care.

The CoC is challenged by multiple aspects such as procedural, administrative, logistics, infrastructural and socio-economic factors therefore restricting LBW infants and their families from receiving adequate postnatal care. Securing the continuity of care minimizes loss to follow-up especially in health systems where linkages between different care levels are weak [44, 79]. Studies from Malawi and Ethiopia have also indicate heterogenous, unstructured postnatal caring systems for LBW infants, which is consistent with the observations made in this study [49, 79] which reflects on how common unstructured postnatal caring systems are in African countries. In Malawi [49], first contacts of LBW infants with community care happened six weeks after birth when families sent them for their first vaccination. Discharge procedures were not optimally implemented due to uncoordinated discharge systems partly attributed to financial and human resource constraints alongside bad phone networks also reported in Ghana [12], Nigeria [67] and Ethiopia [79]. As identified in other studies [19, 51], long distance, transportation challenges, financial constraints, and previous unpleasant encounters at health facilities impede care seeking and a smooth CoC. When care for sick LBW infants is not available in lower-level facilities as it is the case in the CHPS in Ghana, families need to be amply informed on the reasons for referral in order to enhance their willingness to go to the referred facility [79].

Hospital review visits are critical to track the growth and development of LBW infants. A study in Rwanda found high rates of health problems, undernutrition and impaired development in children born LBW and premature [48]. Review visit is an avenue to counsel families on activities to promote growth and development of their LBW infants. But in Sub-Saharan Africa with Ghana being no exception, services that support caregivers to promote positive parenting, stimulate cognitive, improve nutrition, and help high risk children to achieve developmental milestones are frequently missing but are crucial to reduce the impact of impairments [48, 68, 83]. Review visits to health facilities are often challenged by scarcity of resources from both families and health facilities including inadequate health infrastructure [49], insufficient specialists, high staff turnover, and poor communication between the different levels of care [79].

Although our study identified barriers hindering the CoC, it also discovered some enabling activities that facilitate its effective operationalization. Similar to Bazzano et al. [19], community education and knowledge of families on newborn care and ability to detect newborn danger signs were enabling factors. Further, community volunteers are a vital resource to locate LBW infants in the communities where families face difficulty recognizing danger signs or are unable to seek care due to financial issues. In Kenya [55] and Ghana [75], community health volunteers play an important role in early detection of newborn illness and aid prompt referral. The community volunteers’ work in Ghana is hindered by lack of community support [51] but the need for incentives to motivate them is well recognized [18, 75].

Like we found, previous studies have reported on non-availability of evidence-based protocols and guidelines in health facilities targeting preterm, LBW and sick newborns [69, 82]. Where available, policy documents are often poorly disseminated to lower-level managers [22, 62]. Ineffective dissemination and use of outdated materials contribute to health professionals’ non-adherence to protocols and guidelines leading to suboptimum caring practices [62, 82]. Based on the available evidence, it can be argued that frontline workers do not make use of evidence-based guidelines because Ghana Health Service has not effectively implemented certain policies articulated by the Ministry of Health [1, 70]. This is likely due to a combination of factors, including inadequate financial resources and a shortage of human capital [6, 8].

Both the clinical and community-based HCPs emphasized the desire for more supervision as a channel to highlight their challenges, address flaws and improper techniques in the care process. But this supervision mechanism and a system for determining adherence to service delivery standards are often lacking [14, 82] or poorly implemented [22]. Two studies in Ghana showed that staff are motivated by supportive supervision, financial incentives, well-functioning infrastructure and the possibility for continued professional development [40, 53]. In addition, our study revealed that patient/client satisfaction was a motivation for health professionals. Having dedicated, motivated and experienced HCPs to provide quality care for LBW infants is paramount. However, annual staff reshuffling results in knowledge loss, increase in less experienced staff, suboptimum care provision, increased training needs and difficulty adhering to policies and procedures [22, 69, 73]. Meanwhile, in many jurisdictions, well-trained health professionals are missing in almost all levels of health facilities [22, 31, 62] contributing to suboptimal assessment and treatment of LBW infants [81].

Although having a health insurance increases the likelihood of receiving higher quality care during the antenatal, intrapartum and postnatal period [66], challenges in the disbursement of claims and procurement of medical supplies have been reported in studies conducted in Ghana [18, 52]. For example, this situation often resulted in families having to purchase drugs from pharmacies outside the hospital, leading to delays in treatment [5, 18]. Some services for premature infants are also not covered by the current NHIS and therefore the expansion of the current coverage is recommended [5].

Prioritizing preterm and LBW infant care, while simultaneously strengthening communication channels between the different levels of care, and among communities, families and HCPs, can lead to an enhancement of quality of care for LBW infants [47, 82].

### Strengths and limitations

The quality of this study was evaluated using Charmaz’s [25, 26] quality criteria. All the expected criteria were achieved which adds to the strength of the study. Inclusion of health professionals and managers at all levels of care is another asset of the study which facilitated an in-depth understanding of the CoC from different viewpoints. Although not generalizable beyond the study context, findings generated new knowledge that resonates well with existing theories and is transferable to other settings and populations with similar characteristics.

However, it was challenging interviewing the fathers probably due to their busy work schedules, non-availability or unwillingness to participate. Views from stakeholders such as community volunteers, queen mothers and chiefs as well as health managers at national level and politicians could have provided additional insights to complement the CoC model. Hence, research on the above-mentioned stakeholder involvement in the neonatal care period in Ghana is necessary to inform policy and evidence-based guideline development. The coincidence that all LBW infants of our participants were female might have had some implication on the care provision since a particular sex could stimulate families to be concerned with their care.

### Health policy implications

We developed a theoretical model for the CoC of low-birth-weight infants that outlines how the work environment of health professionals can be improved to better support families of LBW infants in the care process. Several studies suggest that policy efforts are warranted to achieve quality of care in order to improve maternal and neonatal outcomes. Through the involvement of families into the care continuum, provision of requisite resources such as financial and human and better coordinated trainings for health professionals, care quality can be improved and progress maintained [7, 22, 34, 74]. Training health care professionals on family systems care can be an avenue to include families of LBW infants more into the care continuum for LBW infants. With a family systems care approach, the autonomy of mothers can be maintained through careful assessment of the family’s structure and gender’s influence, cultural beliefs and who should be with the mother based on her preferences [56, 78, 91]. This can enhance quality of care, reduce tension within the family as well as the financial burden for families and the health system [59, 84, 93].

A coordinated care process and effective communication among health workers in the different levels of care and with families could enhance support for LBW infants. Specifically, when LBW infants are referred, be it for specialist care to a higher facility or community care at the primary level, the referring institution should provide detailed medical history and care instructions and ensure that the referral is completed. Liaising with primary health care services who provide home visitations after discharge has been recommended to improve neonatal health [45, 46, 54, 83]. Community volunteers’ involvement can be strengthened to identify LBW infants not linked with postnatal community care. The important linkages between relevant institutions and the community should be integrated into national programs [83]. Guidelines on discharge and referral processes need to be clearly outlined, distributed and implemented at all levels of care. Such guidelines could guide health workers and promote continuity of care. Stringent, systematic supportive supervision is necessary at all levels of care to prevent HCPs from developing inappropriate practices or making incorrect decisions and to strengthen the use of national guidelines [10, 15, 17, 22]. Implementation research could direct health managers and policy makers on strategies to facilitate effective guideline implementation at all levels of care to achieve well-coordinated and quality care along the CoC for LBW infants.

In order to address this shortfall at the hospital and community levels, it is important to improve the skills of health professionals to use evidenced-based findings of academic and operations research and to translate it into practice [1]. Given that nurses and midwives constitute the largest healthcare workforce globally [88] and contribute substantially to better quality of care, they must be taken into account when formulating guideline implementation policies [76]. Barriers such as insufficient space, limited budgets for commodities, and low staffing can be minimized with well-coordinated investments pivoted on committed leadership and strong political will [47, 62].

## Conclusion

Health professionals recognize the importance of linkages between the different care levels, but a smooth transition within the CoC is hindered by multiple barriers such as procedural, administrative, logistics, infrastructural and socio-economic factors. Expanded coordinated investments into training and decreased staff reshuffling are required to increase the number of specialized neonatal health personnel. Frequent supportive supervision is an avenue to increase work motivation and can steer towards an effective implementation of existing guidelines. Improving quality of the care continuum for LBW newborns requires stakeholder involvement at all levels, strong political will and committed leadership which would invariably reduce resource constraints and enhance the work environment.

### Supplementary Information

**Additional file 1.** Interview Guide and sociodemographic characteristics (parents/family members - home level).

**Additional file 2.** Interview Guide and sociodemogaphic characteristics (health professionals - hospital level).

**Additional file 3.** Interview Guide and sociodemographic characteristics (Health professionals - community level).

**Additional file 4.** Interview Guide and sociodemographic characteristics (Health care planners & coordinators – diff. levels).

## Data Availability

The interview guides used in this study were developed for this study and have never been used before. The datasets analyzed during the current study are available from the corresponding author on reasonable request.

## Abbreviations

BW: Birth weight
CHPS: Community-based Health Planning and Services
CoC: Continuum of Care
ENAP: Every Newborn Action Plan
GA: Gestational age
HCM: Health Care Manager
HCP: Health Care Professional
LBW: Low Birth Weight
MNCH: Maternal, Neonatal and Child Health
NICU: Neonatal Intensive Care Unit
PHNU: Public Health and Nutrition Unit
