# Supplementary material for: Health-system drivers influencing the continuum of care linkages for low-birth-weight infants at the different care levels in Ghana
Source: BMC Pediatr. 2023 Oct 5;23:501. doi: 10.1186/s12887-023-04330-5 (PMC10552361; doi:10.1186/s12887-023-04330-5)
Supplement: Supplementary file 1 — Additional file 1. Interview Guide and sociodemographic characteristics (parents/family members - home level). [file 12887_2023_4330_MOESM1_ESM.pdf]

Interview Guide - Parents/family members – home level

Date of Interview:		Place of Interview:		Urban:	
Start time:		End time:		Rural:	
Name of Interviewer:		File name/ Interview code:			
Name of Translator/RA:		Type of Caregiver:			

Introduction

- Thank participant for participation and time
- Outline aim of study
- Interview duration approximately 60 minutes
- Ask for open questions/concerns
- Emphasise that questions can be asked anytime
- Emphasise that participant can choose not to answer any question(s) which makes them feel uncomfortable
- Emphasise that you will ask a question, will listen, not interrupt until participant has finished talking

Theme: Being a parent/family member of a LBW infant

Invitation to narrate: When having a small baby people typically have some good and some bad days. Some things bring us pleasure, or even joy, and some things we don't like or make us feel miserable. We are interested in both. First, let's talk about the good things.

- Now please tell me about the bad things you experience.
- Challenges/difficult situations since the time of birth

Theme: Time at the MBU

Invitation to narrate I am interested how the time was for you when your baby (your grandchild/niece/nephew etc) was admitted at the MBU. Can you please tell me how you experienced the time in the MBU?

- A (typical) day at the MBU
- People involved in the care
- Good situations/Difficult situations (feelings, impressions)
- Wishes/needs of parents/caregivers

Theme: Time of discharge
**Invitation to narrate:** Before small babies go home parents (other family members) often need to learn a lot about their newborn baby. Can you tell me what was important for you to learn or get to know before you went home?
- Missed instruction/Missed information (from whom)<br>- What was difficult /What was easy
**Theme: First few days at home**
**Invitation to narrate:** You have been home with your small baby for some time now. Being a parent (or family member) of a small baby can bring joy but also responsibilities. Can you please tell me how the time has been for you since you are home with your small baby?
- Can you tell me everything which comes to your mind when you think about the moment when you came home with your small baby?<br>- Provision of care<br>- Fears/Anxiety<br>- Experiencing emergency situations<br>- Challenges/Facilitators<br>- Areas of needing support
**Theme: Community health care**
**Invitation to narrate:** Small babies sometimes need follow up care or fall sick when they are home. I am interested in your experience in seeking care for your small baby. In case you did seek care, can you tell me about that day?
- How was it for you when you first visited a health care centre/health facility?<br>- If you haven't gone to seek health care since you have been discharged from the MBU, can you tell me for what reason you would go and seek health care/help from a person?<br>- Place/facility of seeking care<br>- Alternative care support (Probe for different care providers, including TBA, traditional healers)<br>- Expectations from health professionals<br>- Concerns
**Theme: Barriers/Facilitators**
**Invitation to narrate:** You have experienced a lot through the last few days and weeks since your small baby has been born. I am interested in your opinion. What do you think should be improved in the care for small babies?
- Expectation from health professionals (hospital/community)<br>- During discharge process, what was helpful<br>- Recommendations/Suggestions

**Probing questions**
- That is interesting, can you please tell me more about it?<br>- If you recall, could you tell me how you learned to handle this xxx<br>- Would you tell me how you define it, so I have it in your words?<br>- When you were discussing…..can you tell me how that made you feel?"<br>- You mentioned earlier that…..can you explore that in a little more detail?"<br>- You stated that…..can you explain what you meant by that?"<br>- You said that……..how did that affect you?"
**At the end of the interview**
- Is there something you like to share which I have not ask you about/we have not discussed so far?<br>Is there something you like to ask me?<br><br>- Thank respondent for his time, willingness to participate and sharing his knowledge<br>- Ask respondent if she/he is willing, if required, to be interviewed again at a later stage of the research<br>▪ ☐ Yes ☐ No<br>- Ask participants if she/he/they are interested in the results<br>▪ ☐ Yes ☐ No

**Socio demographics parents/family members**					
**Infant**					
**Date of birth:** (Day/Month/Year)			**Sex of LBW infant:** (Thick appropriate box)	**Female** ☐	**Male** ☐
**Birth weight:**		**g**	**Gestational Age** (If known)	**weeks**	
**Number of children alive:**			**Stillbirth:** (>competed 22weeks GA)		
**Born Normal weight**	**Born Low birth weight**		**Miscarriage:** (<22 weeks GA)*		
**Caregiver**					
**Age:**		**years**	**Gender**	**Female** ☐	**Male** ☐
**Marital status:**	**Married** ☐		**Divorced** ☐	**Single** ☐	**Co-habituating** ☐
**Religion:**	**Christian** ☐	**Muslim** ☐	**Traditionalist** ☐	**Others (specify)**	
**Educational level:**	**Tertiary** ☐	**SHS** ☐	**JHS** ☐	**Primary** ☐	**None** ☐
**Occupation:**	**Employed** ☐	**Unemployed** ☐	**(Specify)**		

If interviewed more than one person – seek information from all participants if they agree

*ICD10
