# Supplementary material for: Health-system drivers influencing the continuum of care linkages for low-birth-weight infants at the different care levels in Ghana
Source: BMC Pediatr. 2023 Oct 5;23:501. doi: 10.1186/s12887-023-04330-5 (PMC10552361; doi:10.1186/s12887-023-04330-5)
Supplement: Supplementary file 2 — Additional file 2. Interview Guide and sociodemogaphic characteristics (health professionals - hospital level). [file 12887_2023_4330_MOESM2_ESM.pdf]

Interview Guide and sociodemographic characteristics (health professionals - hospital)			
Date of Interview:		Place of Interview:	
Start time:		End time:	
Name of Interviewer:		File name/Interview code:	:
Position of Interviewee:			

Introduction

- Thank participant for participation and time
- Outline aim of the study
- Interview duration approximately 60 minutes
- Ask for open questions/concerns
- Emphasise that questions can be asked at anytime
- Emphasise that participant can choose not to answer any question(s) which makes them feel uncomfortable
- Emphasise that you will ask a question, will listen, not interrupt until participant has finished talking

Theme: Experiences working with LBW infants and families in the hospital

Invitation to narrate: Caring for sick babies can be rewarding but at the same time also very challenging. I am interested in your experiences. Can you tell me how it is for you to provide care for LBW infants here in the MBU?

- Challenges/stressors in the care of LBW infants & their families
- Coping technics to overcome stressors
- Parent/family member involvement (family centred care)
- Expectations from authorities/policies

Theme: Discharge of LBW infants

Invitation to narrate: For parents leaving the hospital with a LBW infant can be a joyous but also a bit of a scary, challenging moment. Can you tell me how a normal discharge process looks like?

- People involved in discharge process (parents/caregivers/health professionals/stakeholders)
- Needs/key topics for parent education/counselling
- Guidelines/standards (national, international)
- Referral system/referral letter
- Networking/collaboration with CHPS zones/community health centres
- Opinion, what challenges parents/family members of LBW infants face after discharge

**Theme: Facilitators/Barriers**
**Invitation to narrate:** How is work at the MBU? What works well and where is improvement needed?
- Staff shortage, Supplies, resource allocation<br>- Coping technics<br>- Basic/further education/in-service training<br>- Recommendations for improvements<br>- Expectations from authorities/policies
**General probes**
- Would you tell me how you define it, so I have it in your words?<br>- That is interesting, can you please tell me more about it?<br>- If you recall, could you tell me how you learned to handle this xxx<br>- When you were discussing⋯can you tell me how that made you feel?"<br>- You mentioned earlier that⋯can you explore that in a little more detail?"<br>- You stated that⋯can you explain what you meant by that?"<br>- You said that⋯how did that affect you?"
**At the end of the interview**
- Is there something you like to share which I have not ask you about/we have not discussed so far?<br>- Is there something you like to ask me?<br><br>- Thank respondent for his/her time, willingness to participate and sharing his/her knowledge<br>- Ask respondent if he/she is willing, if required, to be interviewed again at a later stage of the research<br>▪ ☐ Yes ☐ No<br>- Ask participants if he/she is interested in the results<br>▪ ☐ Yes ☐ No

Socio demographics				
**Health professional**				
**Age of health professional:**	**years**	**Sex:**	**Female** ☐	**Male** ☐
**Number of years of working:** (Total years of working)	**years**	**Number of years working in this position/ward:**	**years**	
**Numbers of years working in HMH**		**Previous work experiences** (specify place/date)		
**Educational background:** (specify)		**Year/Place of education:** (specify place/date)		
**Further education:** (eg. Special pediatric/Neonatal care)		**Year/Place of education:** (specify place/date)		
