# Supplementary material for: Health-system drivers influencing the continuum of care linkages for low-birth-weight infants at the different care levels in Ghana
Source: BMC Pediatr. 2023 Oct 5;23:501. doi: 10.1186/s12887-023-04330-5 (PMC10552361; doi:10.1186/s12887-023-04330-5)
Supplement: Supplementary file 3 — Additional file 3. Interview Guide and sociodemographic characteristics (Health professionals - community level). [file 12887_2023_4330_MOESM3_ESM.pdf]

**Interview Guide and sociodemographic characteristics (Health professionals - community level)**					
Date of Interview:		Place of Interview:		Urban:	
Start time:		End time:		Rural:	
Name of Interviewer:			File name/Interview code:	:	
Position of health professional	:				

Introduction

- Thank participant for participation and time
- Outline aim of study
- Interview duration approximately 60 minutes
- Ask for open questions/concerns
- Emphasise that participant can choose not to answer any question(s) which makes them feel uncomfortable
- Emphasise that you will ask a question, will listen, not interrupt until participant has finished talking

Theme: Experiences working with LBW infants and families in the community

Invitation to narrate: Caring for sick and small babies can be rewarding but at the same time also very challenging. I am interested in your experiences. Can you tell me how it is for you to provide care for LBW infants here in this community?

- Typical tasks in the care of LBW infants
- Challenges/stressors in the care of LBW infants & their families
- Parent/family member involvement (family centred care)
- Health professionals/Stakeholder involvement
- Needs/wishes from health professional

Theme: Referral system

Invitation to narrate: When LBW infants come home, they may fall sick or need routine follow up care.. Can you tell me how you get to know that you have a LBW infant (from the HMH) in your community?

- Function of referral system/referral letter
- People involved in discharge process
- Guidelines/standards (local, international)
- Network/collaboration with Hohoe Municipal hospital (higher level facility)
- Opinion, what challenges parents/caregivers of LBW face after discharge

Theme: Facilitators/Barriers

Invitation to narrate: How is it to work as a nurse/ midwife in this community? What works well, where is improvement needed?

- Staff shortage, Supplies, resource allocation
- Coping technics
- Needs
- Expectations from authorities/policies
- Basic/further education/in-service training
- Recommendations for improvements

Probing Questions

- That is interesting, can you please tell me more about it?
- If you recall, could you tell me how you learned to handle this xxx
- Would you tell me how you define it, so I have it in your words?
- When you were discussing……. can you tell me how that made you feel?"
- You mentioned earlier that…….. can you explore that in a little more detail?"
- You stated that……can you explain what you meant by that?"
- You said that………how did that affect you?"

Theme: At the end of the interview

- Is there something you like to share which I have not ask you about/we have not discussed so far?
- Is there something you like to ask me?

- Thank respondent for his/her time, willingness to participate and sharing his/her knowledge
- Ask respondent if he/she is willing, if required, to be interviewed again at a later stage of the research
    - ☐ Yes ☐ No
- Ask participants if he/she is interested in the results
    - ☐ Yes ☐ No

Socio demographics				
**Health Professional**				
**Age of health professional:** (thick appropriate box)	**years**	**Sex:**	**Female** ☐	**Male** ☐
**Number of years of working:** (Total years of working)	**years**	**Number of years working in this position/ward:**	**years**	
**Numbers of years working in HMH** (specify place/years)		**Previous work experiences**		
**Educational background** (specify)		**Year/Place of education** (specify)		
**Further education** (e.g special paediatric/neonatal care)		**Year/place of education:**		
