# Supplementary material for: Health-system drivers influencing the continuum of care linkages for low-birth-weight infants at the different care levels in Ghana
Source: BMC Pediatr. 2023 Oct 5;23:501. doi: 10.1186/s12887-023-04330-5 (PMC10552361; doi:10.1186/s12887-023-04330-5)
Supplement: Supplementary file 4 — Additional file 4. Interview Guide and sociodemographic characteristics (Health care planners & coordinators – diff. levels). [file 12887_2023_4330_MOESM4_ESM.pdf]

Interview Guide and sociodemographic characteristics (Health care planners & coordinators – diff. levels)					
Date of Interview:		Place of Interview:		Urban:	
Start time:		End time:		Rural:	
Name of Interviewer:			File name/Interview code:	:	
Position of health professional		:			

Introduction

- Thank participant for participation and time
- Outline aim of study
- Interview duration approximately 60 minutes
- Ask for open questions/concerns
- Emphasise that questions can be asked anytime
- Emphasise that participant can choose not to answer any question(s) which makes them feel uncomfortable
- Emphasise that you will ask a question, will listen, not interrupt until participant has finished talking

Theme: Involvement in the implementation/planning of LBW infants

Invitation to narrate: Health systems are complex systems involving different services and need a lot of planning and organisation. Can you tell me ways in which you are engaged in the planning and implementation of care for LBW infants respectively at your institution and/or at district/regional level?

- From admission to discharge, at community level, health systems planning, political level
- Job duties
- Financing/Budgeting
- Challenges
- Partners/stakeholder involvement

Theme: Regional and National, international health policies

Invitation to narrate: Organisation, planning and implementation of health systems and health services often follow guidelines and specific policies. Can you tell me if there are any regulations and guidelines that in your opinion play an important role in the planning and implementation of care for LBW infants?

- Regional and national policies and guidelines (probe for ENAP, CoC, Universal health for all)
- Programmes (focus on Maternal- and Newborn care)
- Incorporation of guidelines between different levels of care
- Own vision/mission in facility/institution in terms of maternal/newborn care
- Knowledge resource, education

Theme: Continuum of Care

Invitation to narrate: The patient pathway for Mothers and LBW infants involves hospitals, community and home care services. How do you experience their transition? What are important interfaces between these different care delivery levels?

- Linkages between the HMH/CHPS compounds/community health
- Challenges in the transition/discharge process of LBW infants
- Other people involved in the care/planning of LBW infants at all levels of care, health policies, politics etc
- Challenges at facility level/community level/Regional/National
- Facilitators
- Recommendations to overcome challenges
- Needs/wishes from Ghana health service/other authorities
- Specific support desire

Theme: Health care professionals &Training of staff

Invitation to narrate: Health care professionals including nurses, midwives and paediatricians/neonatologists are a key factor for good quality care for LBW infants. What can you tell me about health care professionals in the Hohoe Municipal Hospital and your community and their training? (Regionally/nationally)

- Health care professional/patient ratio
- Basic education
- Further/specialised training (Hospital/Community care level)
- In-service trainings

Probing questions

- Would you tell me how you define it, so I have it in your words?
- That is interesting, can you please tell me more about it?
- If you recall, could you tell me how you learned to handle this xxx
- When you were discussing…can you tell me how that made you feel?”
- You mentioned earlier that…can you explore that in a little more detail?”
- You stated that…can you explain what you meant by that?”
- You said that…how did that affect you?”

At the end of the interview

- Is there something you like to share which I have not ask you about/we have not discussed so far?
- Is there something you like to ask me?

- Thank respondent for his/her time, willingness to participate and sharing his/her knowledge
- Ask respondent if he/she is willing, if required, to be interviewed again at a later stage of the research
  - ☐ Yes ☐ No
- Ask participants if he/she is interested in the results
  - ☐ Yes ☐ No

**Socio demographics**				
**Participant**				
**Age of participant:**	**years**	**Sex:**<br>(thick appropriate box)	**Female**<br>☐	**Male**<br>☐
**Number of years of working:**<br>(Total years of working)	**years**	**Number of years working in this position:**	**years**	
**Previous work experiences:**<br>(specify place/date)				
**Educational background:**<br>(specify)		**Year/Place of education:**<br>(specify place/date)		
**Further education:**<br>(specify place/date)		**Year/Place of education:**<br>(specify place/date)		
